# Prospective Short-Term and Return-to-Sports Results of a Novel Uncemented Short-Stem Hip Prosthesis with Metaphyseal Anchorage

**DOI:** 10.3390/jcm9061972

**Published:** 2020-06-24

**Authors:** Robert Breuer, Rainer Fiala, Nina Schrenk, Thomas M. Tiefenboeck

**Affiliations:** 1 Department of Orthopedics and Trauma Surgery, Medical University of Vienna, 1090 Vienna, Austria; robert.breuer@meduniwien.ac.at; 2 Department of Orthopedics and Trauma Surgery, Sozialmedizinisches Zentrum Ost, 1220 Vienna, Austria; rwf3a@virginia.edu; 3 Department of Orthopedics, Klinikum Wels-Grieskirchen, 4600 Wels, Austria; Nina.Schrenk@klinikum-wegr.at

**Keywords:** total hip arthroplasty, short stem, minimally invasive, bone stock preservation, return to sports, physical activity

## Abstract

Short-stem hip prostheses were developed to treat active patients requiring total hip arthroplasty (THA). This study provides short-term data about a short-stem total hip arthroplasty system. Functional and radiological outcomes as well as return to sports and activity level were assessed. A series of 55 patients was primarily included. Data were available for 47 patients at an average follow-up of 38 ± 4.6 months. The back-to-sports analysis showed a 98% return-to-sports rate (46/47 patients). The average time for return to sports was 13 weeks (± 8) postoperatively. Five patients (10.6%) were more active postoperatively. The Harris Hip Score (HHS) improved from 34.8 (±9.4) preoperatively to 94.7 (±8.4, *p* ≤ 0.001) and the University of California, Los Angeles (UCLA) score improved from 4.5 (±1.8) to 6.9 (±1.9) (*p* ≤ 0.001). The High Activity Arthroplasty Score (HAAS) was 12 (±3.6) at 3-year follow-up. Pre- and postoperative UCLA and postoperative HHS and HAAS scores had a positive influence on the return-to-sports rate (*p* ≤ 0.05). The collection of radiographic data during all postoperative follow-ups showed no signs of radiolucent lines or bone fissures. The complication rate was at 5%. Short-stem systems are equaling conventional prostheses and offer benefits regarding soft tissue and bone stock preservation. Fast recovery and return to sports can be achieved.

## 1. Introduction

With the increasing number of young and active patients who meet the indication for total hip arthroplasty (THA) due to osteoarthritis of the hip joint, as well as precipitating pathologies such as post-traumatic osteoarthritis, avascular necrosis, hip dysplasia and rheumatoid arthritis [1,2,3], minimally invasive and muscle-sparing surgical approaches for THA are being constantly enhanced. In consideration of this emerging necessity, short-stem prostheses have been introduced for improved biomechanical reconstruction in the proximal femur, reduced stress shielding, and bone preservation in case of revision surgery at a later time [4]. Short stems engage within either the femoral neck, lateral cortex, or lateral trochanteric flare [5]. So far, there have been many studies about primary total hip arthroplasty using a variety of short-stem prostheses, but there are few data concerning survival or revision rates [6,7,8]. Furthermore, only a few studies exist about the return to physical activity after short-stem hip arthroplasty [9,10]. With the increasing awareness of social and health benefits from regular physical activity, as well as rising expectations of postoperative sports participation, the need for “return-to-sports” education and recommendations should be addressed. 

Therefore, the purpose of this study was to present prospective data on a short stem with metaphyseal anchorage at a follow-up of 3 years with special focus on return to sports and postoperative physical activity level. 

## 2. Experimental Section

### 2.1. Patients

For this prospective observational study, a consecutive series of 55 patients, meeting participation criteria, underwent an operation between March 2015 and July 2016. This study was approved by the ethical committee and received a positive vote (Ethics Committee of Upper Austria, vote B-13617). Full written consent was obtained from every subject before inclusion. Inclusion criteria included severe primary osteoarthritis (severe impairment of activities of daily living, Kellgren–Lawrence Score > 2) of the hip joint after failed conservative management, avascular necrosis of the femoral head, dysplastic osteoarthritis, and age between 18 and 80 years. Exclusion criteria included severe hip dysplasia (Crowe > II), pelvic obliquity (with a highly developed leg length inequality, of more than 2 cm clinically and radiologically), progressive idiopathic scoliosis (with a Cobb angle >50°), neurological disorders, poor bone stock (DEXA T-score < −2), malignant diseases, rheumatoid arthritis, prior fracture of ipsilateral femur or tibia or other lower-extremity femoral/tibial osseous deformity (e.g., coxa valga > 145° or coxa vara < 125°), pregnancy, insulin-dependent diabetes mellitus, and preoperative anemia (hemoglobin < 11 mg/dL). An overview of the patient demographics is outlined in Table 1.

### 2.2. Clinical Examination, X-ray, and Scores

Pre- and postoperative clinical examinations included measurement of range of motion (ROM) in every degree of freedom of the hip joint, evaluation of leg length difference and pelvic obliquity, tendency for dislocation in extension and 90 degrees of flexion, and pain after applying axial load or rocking. Harris Hip Score (HHS) [11], University of California, Los Angeles (UCLA) activity score, High Activity Arthroplasty Score (HAAS), and information about the kind of physical activity and sports were collected preoperatively and at the 3-year follow-up. To bypass the ceiling effect of the Harris Hip Score with respect to more demanding physical activity, the High Activity Arthroplasty Score [12] and UCLA activity score [13,14] represent appropriate measures of activity in THA patients. Radiographs were taken preoperatively and postoperatively after 1 day, 3 months, 6 months, 1-year follow-up, and 3-year follow-up. The x-ray series included pelvic overview and hip a.p. and axial pictures. Patients were screened for signs of radiographic loosening, implant failure, or fissures and fractures. Intraoperative blood loss was calculated using the formula of Bourke [15]. Additionally, operating time and length of hospital stay were measured. 

### 2.3. Surgical Technique and Follow-Up Treatment

**Figure 1:**
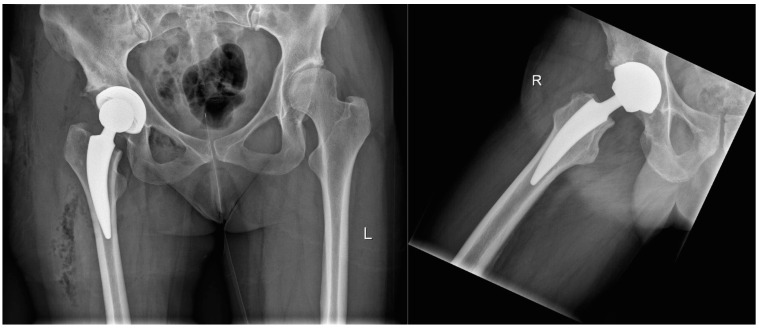
Immediate postoperative situation after MiniMIS implantation on the right site, radiograph a.p. and axial.

The patient is placed in supine position. The minimally invasive Watson-Jones approach is utilized for reduced soft-tissue damage [16]. The capsule is split but not resected. After the femoral neck preserving osteotomy and preparation of the acetabulum, the original cup (Sphaericon^®^, Falcon Medical, Millbury, MA, USA) is implanted according to press-fit surgical protocol. Next, the femoral canal is prepared using specialized, curved rasps, and the correctly sized uncemented stem (MiniMIS^®^, Falcon Medical, Millbury, MA, USA) is implanted. The MiniMIS^®^ short stem is made of a titanium–plasma alloy, covered with calcium phosphate (Bonit^®^) and is available in 10 different sizes. Figure 1 shows a postoperative x-ray to visualize the design (Figure 1). The choice of articular bearing is left up to the surgeon. It can be selected out of ceramic, standard polyethylene, or highly cross-linked polyethylene, depending on the age of the patient. No drainage is used due to meticulous hemostasis. One gram of Tranexamic acid is administered before skin incision. After proper suture of the capsule, as well as subcutaneous and intracutaneous sutures, standard postoperative protocol includes full weight bearing starting at the first postoperative day under supervision of the physical therapy department. The aid of crutches and accompanied hypocoagulation is recommended for 4–6 weeks. 

### 2.4. Statistics

Descriptive data are presented as arithmetic mean and standard deviation unless stated otherwise. Comparisons between pre- and postoperative levels of Harris Hip Score (HHS), UCLA score, hemoglobin, and sport disciplines were calculated using paired *t*-tests. To examine whether criterion variables (age, gender, body mass index (BMI), ASA (American Society of Anesthesiologists) score [17], HHS, HAAS, UCLA) predicted the return-to-sports ratio, subsequent logistic regression models were calculated instead of a joint model, because of the fair sample size to avoid potential power-related bias. Return to sports was dichotomized (0 = no return, 1 = return). To examine whether criterion variables (age, gender, BMI, ASA score, HHS, HAAS, UCLA) predicted the time it took participants to return to sports, subsequent linear regression models for all participants who were eligible (return to sports = 1) were calculated. Potential differences between male and female participants regarding the time to return to sports and postoperative levels of HHS, UCLA, and HAAS were calculated using independent *t*-tests. The level of significance for all conducted analysis was set to *p ≤* 0.05. Statistical analyses were performed using SPSS 26.0 (SPP Inc., Chicago, IL, USA).

## 3. Results

We report on the outcome of 55 MiniMIS^®^ (Falcon Medical) short stems with average follow-up of 38 months (±4.6). Of these initial 55 patients, we had a loss of eight patients at the latest follow-up (14.5%). Three patients died during the follow-up period (one due to carcinoma, one due to pneumonia, one due to brain ischemic insult). Three more patients withdrew their previously given consent for participation and two patients could not be traced, resulting in a final sample of 47 patients (85%) voluntarily taking part in the present study at the time of follow-up. An overview of the outcome parameters can be found in Table 1 and Table 2.

Length of hospital stay averaged 7.5 (±2.2) days (Table 2). The average preoperative hemoglobin level was 14.7 g/dL (±1.5) and it significantly decreased to 11.1 g/dL (±1.7) on the first day post-OP (*p* ≤ 0.001). Calculated blood loss was at 1098 mL (±785). Three of the 55 initially operated patients (5%) required blood transfusions postoperatively after showing a hemoglobin drop below 8 mg/dl. The average body mass index (BMI) was 27.9 ± 4.9 kg/m^2^. We could not find any significant influence of surgery time on blood loss (β = 0.035, *p* = 0.82). Furthermore, intraoperative blood loss did not significantly affect duration of hospital stay (β = 0.047, *p* = 0.76). 

Table 3 provides a comparison to other implants and surgical approaches (Table 3).

### 3.1. Functional Results and Return to Sports

Looking at functional capability of the patients, the Harris Hip Score (HHS) improved from 34.8 (±9.4) preoperatively to 94.7 (±8.4) postoperatively at the 3-year follow-up (*p* ≤ 0.001). The UCLA activity score increased significantly from 4.5 (±1.8) to 6.9 (±1.9) (*p* ≤ 0.001). The High Activity Arthroplasty Score (HAAS) was 12 (±3.6) at 3-year follow-up. Male patients reached significantly higher scores than female patients in all postoperatively measured scores, including HHS (*p* ≤ 0.001), HAAS (*p* ≤ 0.001), and UCLA (*p* ≤ 0.01). The back-to-sports analysis demonstrated a 98% return-to-sports rate, with 89% (42/47 patients) returning to their respective preoperatively performed physical activities, and an additional four patients (9%) starting sports postoperatively for their first time. In addition, 27/47 patients (57.4%) were able to successfully return to all physical activities practiced before the onset of surgery-demanding symptoms. On the other hand, 8/47 patients (17.1%) were forced to stop certain activities because of other causes such as coronary artery disease, general dizziness, age, or pain within other body segments. Furthermore, 7/47 patients (14.9%) reported hip-related limitations (pain, limitation of ROM) in certain activities; however, only one patient needed to stop physical activity because of pain and stiffness. One female patient was not able to participate in physical activity because of a persistent intraoperative lesion of the femoral nerve. The other primarily mentioned main causes were anxiety/fear of injury and physicians’ discouragement. Interestingly, 5/47 patients (10.6%) reported being more active postoperatively than ever before. Ninety percent of all patients could return to sports within the first six months after surgery. The average time for returning to physical activity postoperatively was 13 (±8) weeks (Table 2). In total, the patients were engaging in an average of 1.89 (±1.1) different sport disciplines before surgery, which non significantly decreased to 1.7 (±1.1) postoperatively (*p* = 0.162) (Table 4). Most of the patients performed a mix of low- and high-impact sports with a focus on low-impact and recreational activities such as cycling, swimming, and Nordic walking. Sole high-impact sports such as tennis, downhill skiing, or soccer were performed by eight patients preoperatively before the onset of symptoms, and were continued by five patients postoperatively (Table 5).

A significant influence of age on the return-to-sports rate could not be detected, but a trend was found that older patients returned less commonly to their sports (OR = 0.88, *p* = 0.07). Interestingly, age did have an inverse effect on the time patients took to return to sports (*p* ≤ 0.05). Younger patients took significantly longer to return than older patients (β = −0.33, *p* ≤ 0.05). 

In contrast, neither preoperative score values of UCLA and HHS (*p* = 0.643) nor follow-up score values of UCLA, HHS, or HAAS (*p* = 0.432) had any significant influence on the time interval until return to sports. Likewise, preoperative HHS (*p* = 0.425) did not significantly predict the return-to-sports rate. 

However, preoperative UCLA score (*p* ≤ 0.05, OR = 2.60), follow-up UCLA score (*p* ≤ 0.05, OR = 1.80), follow-up HHS score (*p* ≤ 0.05, OR = 1.10), and HAAS score (*p* ≤ 0.01, OR = 1.71) all significantly increased the odds of returning to sports.

BMI (*p* = 0.464) and sex (*p* = 0.746) did not predict the return-to-sports rate, whereas an increased ASA score decreased the odds of returning to sports (*p* ≤ 0.05, OR = 0.87). 

### 3.2. Radiographic Results and Complications

The collection of radiographic data at the first preoperative day (antero-posterior view) and during postoperative follow-up visits (antero-posterior and lateral views) at 3 months, 6 months, 12 months, and 36 months showed no signs for radiolucent lines, bone fissures, or implant failure (Figure 1). One patient falling ill with pneumonia on the fifth postoperative day recovered quickly after instillation of intravenous antibiotics, being able to leave the hospital in well general condition two weeks postoperatively. One lesion of the femoral nerve was recorded at follow-up. There were no local infections, but one revision was required after traumatic (non-sports related) periprosthetic femoral fracture four weeks postoperatively. 

## 4. Discussion

The MiniMIS^®^ short stem was developed with a neck-preserving design to enable a bone sparing operational procedure in total hip arthroplasty, being achieved by metaphyseal anchorage within the load bearing area of the femoral neck. This allows for minimally invasive surgical techniques and consecutively maximal bone and tissue preservation.

As can be seen in the perioperative parameters, our series can be well compared to other papers regarding short-stem prostheses (Table 3). Interestingly, blood loss seems to be higher after surgery in Europe in comparison to the rest of the world [19]. We tried to find a reason for this detail, but we were not able to identify a cause. There are many different methods to calculate intraoperative blood loss, and most measurements are done by indirect calculations. Nevertheless, we could confirm previous findings about lower blood loss and decreased transfusion rates after short-stem THA with a minimally invasive approach in comparison to conventional stems, where transfusion rates of up to 70% are reported [19]. Besides the risk of transfusions, it is also a very important economic factor. 

It is evident that there is a certain learning curve regarding proper sizing and positioning of the short stem, especially for young and inexperienced surgeons [24]. Nevertheless, our study shows that operation times similar to total hip arthroplasty with conventional stems can be achieved by qualified surgeons. Yu et al. [25] described average surgical times of 67.4 ± 5.8 min for short-stem implantation and 69.5 ± 7.8 min for conventional stems, which are well comparable to our average operative time of 67.6 ± 14.0 min and to other short-stem implants (Table 3).

Our observed average duration of hospital stay of 7.9 ± 2.2 days after total hip arthroplasty with short-stem implantation correlates well with acceptable standards in the current literature, showing rapid recovery [26,27]. As can be seen in the overview, even shorter hospital stays can be reached via fast track surgery concepts. 

Another positive finding was the complete lack of local infections, which is being cited in the literature as 2.5% for surgical site infections and 0.9% for deep joint infections [28]. Additionally, only one revision surgery had to be undertaken within the mentioned follow-up period, resulting from a non-sport related stumble in the fourth postoperative week. The literature describes an overall rate of intraoperative and early postoperative periprosthetic fracture of 0.1% to 27.8% in conventional total hip arthroplasty [29,30]. Furthermore, we did not observe any radiographic signs of implant failure or loosening, which matches previously reported data of similar short-stem systems [31,32]. 

The overview shows higher complication rates in studies in which a direct anterior approach was chosen (Table 3). This is consistent with findings in the literature, especially in contrast to posterior approaches [33,34]. Inconsistent data exist when comparing that approach to the anterolateral one [35]. In our series, however, we were able to reach an overall complication rate comparable to the posterior approach. 

A similar functional outcome compared to conventional stem implants may be reached using the MiniMIS^®^ short stem. Pogliacomi et al. [19] demonstrated a mean Harris Hip Score of 91 for the implantation of conventional stems using the direct anterior approach, and a score of 89 for the lateral approach at one-year follow-up. A study by Stadler et al. [32], using the Nanos^®^ short stem, described postoperative Harris Hip Scores of 94.5 ± 8.8 at one-year follow-up. Using the anterolateral approach, we were able to show comparable postoperative Harris Hip Scores at 3-year follow-up of 94.7 ± 7.1.

There have been a few publications concerning return-to-sports assessment after conventional stem total hip arthroplasty. Within a retrospective cohort study by Innmann et al., 89% of preoperatively active patients (*n* = 86, mean age 52 years at surgery) were able to return to sports after a 10-year follow-up period [36]. Similar results were shown in a registry-based long-term study by Lübbeke et al. [37]. To our knowledge, there have only been two studies to sufficiently document return-to-sports data after short-stem THA, showing return-to-sports rates of 91% (114/126 patients) at minimum follow-up of 20 months, and 98% (77/79 patients) at minimum follow-up of 24 months [9,10]. However, all of these studies are of retrospective design. The above-mentioned return-to-sports rates are comparable to our 98% rate (46/47 patients). Despite the significant decrease, our UCLA scores of 7.49 before symptom onset and of 6.87 at 3-year follow-up show competitive results compared to conventional stem THA and the mentioned studies about short-stem THA (Table 4). The raised value also shows a close relation to the threshold of intense physical activity mentioned above and, in absolute numbers, a fairly high quantity of the patients could still exercise at a very high level (UCLA > 7). Our High Activity Arthroplasty Score of 11.95 also matches the results of Talbot et al., reporting a score of 11.25 at 4-year follow-up in a sample of 99 patients aged < 66 years [12]. We found a similar return-to-sports time interval and a similar number of sports disciplines (Table 4). 

We tried to identify influencing factors on the return-to-sports rate or the time interval until the patients were able to return to sports, but could not show any conclusive results for the time interval. This is probably because of the relatively low number of patients in our series. Nevertheless, we could show that higher postoperative score values increased the possibility of patients returning to their sports, which seems to be obligatory since all the scores are measuring physical activity. However, solely a higher preoperative UCLA score likewise increased the chance of returning to sports, whereas the preoperative Harris Hip Score did not have any influence on the back-to-sports rate. A higher ASA score, as an indicator for comorbidity, decreased the possibility of return to sports, which seems reasonable as well. However, studies with a higher number of participants must be conducted in order to determine the prediction value of our findings.

Concerns have been raised that higher levels of physical activity may increase the risk of dislocation, fracture, or poor outcome after total hip arthroplasty, but no clear evidence yet exists [38]. There is potentially some trade-off between the patients’ desire to pursue certain high-impact activities and the risk of raised wear rates [39]. The comparatively high cycling loads can lead to increased wear in certain bearing couples, especially when using conventional polyethylene. Wear rates can be reduced by using more advanced coupling such as ceramic/ceramic or ceramic/highly crosslinked polyethylene [40]. We have seen no evidence that high levels of sporting activity or high-impact sports lead to poor clinical or radiological outcome at least three years postoperatively. 

Current recommendations for physical activity after total hip arthroplasty still favor low-impact activities such as swimming, walking, cycling, and golf to an “unlimited” extent, but high-impact activities such as jogging, downhill skiing, and singles tennis should be “discouraged” [39,41]. Delasotta et al. found that the majority of their surveyed patients participated in recommended activities, where the main reasons for not resuming beloved high-impact activities were fear of injury (28.6%) and physician recommendation (25.7%) [41]. Abe et al. showed that jogging, as a high-impact activity, has no negative influence at least as a short-term outcome. Furthermore, they pointed out that pain, early fatigue, or lack of interest were not the primary reasons for stopping high-impact activities [38]. Nevertheless, there are still no long-term results regarding increased wear rates with modern coupling systems when performing high-impact sports [42]. These findings correlate with our results, where 14.9% of the patients did not return to their preoperative high-impact activities due to the above-mentioned reasons. Another 17.1% felt impaired in their ability to pursue certain physical activities due to other health-related causes. However, we observed 10.6% achieving a higher level of activeness postoperatively, mainly because of instilled behavioral changes with perceived functional improvements during rehabilitation/postoperative physical therapy. In our own approach, we recommended standardized rehabilitation consisting of physiotherapy and controlled non-weight bearing activities for the first three months. Then, the clearance was given for low-impact sports depending on the patient’s personal situation and possible impairment. We did not encourage high-impact sports before six months postoperatively. Nevertheless, there is no clear evidence for an optimal timeframe regarding the return to full activity after THA.

The major strength of our study was, of course, the prospective study design, which made it the first prospective study to evaluate back to sports after short-stem THA. Furthermore, the homogenous patient collective allowed us to minimize selection bias. 

As a limitation for our findings, our short follow-up period must of course be mentioned. Nevertheless, it was our main goal to assess the time and rate of return to physical activity. Because a short period of time to regain the previous level of activity is desirable, we decided to execute a short-term follow-up. Furthermore, our comparatively young and healthy test population must be noted. However, it should be kept in mind that the indication for total hip arthroplasty with short stem mainly targets this population. Finally, our series was comparatively small, but we introduced a novel short-stem system in our hospital and an evaluation was planned to decide about further use. The introduction of a control group will be our future endeavor. 

The acceptable clinical and radiographic outcomes encourage the usage of short stems with metaphyseal anchorage for total hip arthroplasty. Further studies are required to collect data concerning long-term bone remodeling and the survival rate of femoral neck-preserving implants, integrating possible premature failure resulting from continued high-impact physical activity.

## 5. Conclusions

Short-stem hip arthroplasty is an advanced way of preserving bone stock while protecting soft tissue. This not only allows for a rapid recovery and a satisfactory return-to-sports rate after surgery, but it is also the keystone for a simplified revision surgery after implant failure for the future.

## Figures and Tables

**Table 1:** Patient characteristics. Values are depicted as absolute numbers and percentage unless marked otherwise. ° values depicted as mean and standard deviation, * values depicted as median and range. THA (Total Hip Arthroplasty); ASA (American Society of Anesthesiologists).

Patient Characteristics	
Number of Patients	*n* = 55
Age (y)	61 (±10) °
Sex (m/f)	29/26 (53%/47%)
Reason for THA	Primary Osteoarthritis *n* = 49 (89%)
	Avascular Necrosis *n* = 3 (5.5%)
	Mild Dysplasia *n* = 3 (5.5%)
Follow-up (mo)	38 (±4.6) °
Loss to follow-up	*n* = 8 (14.5%)
Previous hip injuries, surgery, deformities	none
ASA	2 (1–3) *

**Table 2:** Outcome parameters. Values are depicted as arithmetic mean and standard deviation unless marked otherwise.

Outcome Parameters			
	Pre-OP	Post-OP	*p*-Value
Operation time (min)	-	67.6 (±14.0)	-
Hb (g/dL)	14.7 (±1.5)	11.1 g/dl	*p* ≤ 0.001
Blood loss (mL)	-	1098	-
Transfusions (%)	-	5%	-
Hospital stay (d)	-	7.5 (±2.2)	-
Complications (%)	-	5%	-
Sport disciplines	1.89 (±1.1)	1.7 ± 1.1	*p* = 0.162
Return to sports (%)	-	98%	-
Interval until return (w)	-	13 (±8)	-
HHS	34.8 (±9.4)	94.7 (±8.4)	*p* ≤ 0.001
HAAS	-	12 (±3.6)	-
UCLA	4.5 (±1.8)	6.9 (±1.9)	*p* ≤ 0.001

**Table 3:** Perioperative parameters after short-stem total hip arthroplasty. Overview of current literature. Values are depicted as arithmetic mean and standard deviation unless marked otherwise. * Values depicted as median and range.

Perioperative Parameters after Short-Stem Total Hip Arthroplasty							
Study	Surgical Approach	Implant	Blood Loss (ml)	Transfusion Percentage (%)	Operation Time (min)	Hospital Stay (d)	Complications (%)
Ogonda et al. [18]	Posterior (mini-incision)	Xpress Rapid Custom (DePuy)	314	n.r.	60.3 ± 9.2	3.6	3%
Hochreiter et al. [19]	Anterolateral	Optimys (Mathys)	1139	8%	63 (45–91)	n.r.	n.r.
Bernasek et al. [20]	Anterolateral	Summit (Depuy)	357	n.r.	62	3.4	4%
Zhao et al. [21]	Direct anterior	no stated (short stem)	166	8%	83.26 (±6.69)	2.8 (±0.16)	n.r.
Cheng et al. [22]	Direct anterior	Anthology (Smith and Nephew)	n.r.	8.5%	125 (111–138) *	4 (3.1–5.3) *	11%
Barrett et al. [23]	Direct anterior	Corail (DePuy)	391	n.r.	84.3 (±12.4)	2.28	23%
Breuer et al. (this study)	Anterolateral	MiniMIS (Falcon Medical)	1098	5%	67.6 (±14.0)	7.5 (±2.2)	5%

**Table 4:** Functional outcome and back to sports after short-stem total hip arthroplasty. Overview of current literature. Values are depicted as arithmetic mean and standard deviation unless marked otherwise. * Values depicted as median and range.

Functional Outcome and Back to Sports after Short-Stem Total Hip Arthroplasty							
Study	Implant	Return to Sports (%)	Interval Until Return to Sports	Sport Disciplines	Harris/Oxford Hip Score	HAAS	UCLA
Ortmaier et al. [9]	Optimys; (Mathys)	91	<6 Months (87%)	2.6 ± 1.9	45.1 (34–48) *	n.r.	7.1 (4–10) *
Schmidutz et al. [10]	Metha; (BBraun Aesculap)	98	3–6 Months (70%)	3.5 ± 2	93.6 (±6.3)	n.r.	7.6 ± (1.9)
Breuer et al. (this study)	MiniMIS	98	<6 Months (90%)	1.7 ± 1.1	94.7 (±8.4)	12 (±3.6)	6.9 (±1.9)

**Table 5:** High- or low-impact sports. Overview of patients performing high- or low-impact activities or a mix of both kinds of sports.

High- or Low-Impact Sports				
	High Impact	Low Impact	Mixed	No
Pre-OP	*n* = 8 (17%)	*n* = 13 (28%)	*n* = 20 (43%)	*n* = 6 (12%)
Post-OP	*n* = 5 (11%)	*n* = 22 (47%)	*n* = 16 (34%)	*n* = 4 (8%)

## References

[1] Adelani M.A., Keeney J.A., Palisch A., Fowler S.A., Clohisy J.C. (2013). Has total hip arthroplasty in patients 30 years or younger improved? A systematic review. Hip Clin. Orthop. Relat. Res..

[2] McElroy M.J., Johnson A.J., Mont M.A., Bonutti P.M. (2011). Short and standard stem prostheses are both viable options for minimally invasive total hip arthroplasty. Bull. NYU Hosp. Jt. Dis..

[3] Ravi B., Croxford R., Reichmann W.M., Losina E., Katz J.N., Hawker G.A. (2012). The changing demographics of total joint arthroplasty recipients in the United States and Ontario from 2001 to 2007. Best Pract. Res. Clin. Rheumatol..

[4] Huo S.C., Wang F., Dong L.J., Wei W., Zeng J.Q., Huang H.X., Han Q.M., Duan R.Q. (2016). Short-Stem Prostheses in Primary Total Hip Arthroplasty: A Meta-Analysis of Randomized Controlled Trials. Medicine.

[5] Learmonth I.D. (2009). Conservative stems in total hip replacement. Hip Int..

[6] Amenabar T., Marimuthu K., Hawdon G., Gildone A., McMahon S. (2015). Total hip arthroplasty using a short-stem prosthesis: Restoration of hip anatomy. J. Orthop. Surg..

[7] Salemyr M., Muren O., Ahl T., Bodén H., Eisler T., Stark A., Sköldenberg O. (2015). Lower periprosthetic bone loss and good fixation of an ultra-short stem compared to a conventional stem in uncemented total hip arthroplasty. Acta Orthop..

[8] Choi Y.W., Kim S. (2016). The Short-term Clinical Outcome of Total Hip Arthroplasty Using Short Metaphyseal Loading Femoral Stem. Hip Pelvis.

[9] Ortmaier R., Pichler H., Hitzl W., Emmanuel K., Mattiassich G., Plachel F., Hochreiter J. (2019). Return to Sport after Short-Stem Total Hip Arthroplasty. Clin. J. Sport Med..

[10] Schmidutz F., Grote S., Pietschmann M., Weber P., Mazoochian F., Fottner A., Jansson V. (2012). Sports activity after short-stem hip arthroplasty. Am. J. Sports Med..

[11] Harris W. (1969). Traumatic arthritis of the hip after dislocation and acetabular fractures: Treatment by mold arthroplasty. An end-result study using a new method of result evaluation. J. Bone Jt. Surg. Am..

[12] Talbot S., Hooper G., Stokes A., Zordan R. (2010). Use of a New High-Activity Arthroplasty Score to Assess Function of Young Patients With Total Hip or Knee Arthroplasty. J. Arthroplast..

[13] Terwee C.B., Bouwmeester W., van Elsland S.L., de Vet H.C.W., Dekker J. (2011). Instruments to assess physical activity in patients with osteoarthritis of the hip or knee: A systematic review of measurement properties. Osteoarthr. Cartil..

[14] Naal F.D., Impellizzeri F.M., Leunig M. (2009). Which is the best activity rating scale for patients undergoing total joint arthroplasty?. Clin. Orthop. Relat. Res..

[15] Bourke D., Smith T. (1974). Estimating allowable hemodilution. Anesthesiology.

[16] Austin M.S., Rothman R.H. (2009). Acetabular Orientation Anterolateral Approach in the Supine Position. Clin. Orthop. Relat. Res..

[17] Saklad M. (1941). Grading of patients for surgical procedures. Anesthesiology.

[18] Ogonda L., Wilson R., Archbold P., Lawlor M., Humphreys P., O’Brien S., Beverland D. (2005). A Minimal-Incision Technique in Total Hip Arthroplasty Does Not Improve Early Postoperative Outcomes. J. Bone Jt. Surg..

[19] Hochreiter J., Hejkrlik W., Emmanuel K., Hitzl W., Ortmaier R. (2017). Blood loss and transfusion rate in short stem hip arthroplasty. A comparative study. Int. Orthop..

[20] Bernasek T.L., Lee W.L.H., Yang J.L.K.K.J. (2010). Minimally invasive primary THA: Anterolateral intermuscular approach versus lateral transmuscular approach. Arch. Orthop. Trauma Surg..

[21] Zhao H., Kang P., Xia Y., Shi X. (2017). Comparison of Early Functional Recovery after Total Hip Arthroplasty Using a Direct Anterior or Posterolateral Approach: A Randomized Controlled Trial. J. Arthroplast..

[22] Cheng T.E., Wallis J.A., Taylor N.F., Holden C.T., Marks P., Smith C.L., Armstrong M.S., Singh P.J. (2017). Primary Arthroplasty A Prospective Randomized Clinical Trial in Total Hip Arthroplasty d Comparing Early Results Between the Direct Anterior Approach and the Posterior Approach. J. Arthroplast..

[23] Barrett W.P., Turner S.E., Leopold J.P. (2013). Prospective Randomized Study of Direct Anterior vs Postero-Lateral Approach for Total Hip Arthroplasty. J. Arthroplast..

[24] Loweg L., Kutzner K.P., Trost M., Hechtner M., Drees P., Pfeil J., Schneider M. (2018). The learning curve in short-stem THA: Influence of the surgeon’s experience on intraoperative adjustments due to intraoperative radiography. Eur. J. Orthop. Surg. Traumatol..

[25] Yu H., Liu H., Jia M., Hu Y., Zhang Y. (2016). A comparison of a short versus a conventional femoral cementless stem in total hip arthroplasty in patients 70 years and older. J. Orthop. Surg. Res..

[26] Abbas K., Umer M., Qadir I., Zaheer J., ur Rashid H. (2011). Predictors of length of hospital stay after total hip replacement. J. Orthop. Surg..

[27] Olthof M., Stevens M., Bulstra S.K., Van den Akker-Scheek I. (2014). The association between comorbidity and length of hospital stay and costs in total hip arthroplasty patients: A systematic review. J. Arthroplast..

[28] Lindeque B., Hartman Z., Noshchenko A., Cruse M. (2014). Infection after Primary Total Hip Arthroplasty. Orthopedics.

[29] Park K.J., Menendez M.E., Barnes C.L. (2017). Perioperative Periprosthetic Fractures Associated With Primary Total Hip Arthroplasty. J. Arthroplast..

[30] Sidler-Maier C.C., Waddell J.P. (2015). Incidence and predisposing factors of periprosthetic proximal femoral fractures: A literature review. Int. Orthop..

[31] Wacha H., Domsel G., Herrmann E. (2018). Long-term follow-up of 1217 consecutive short-stem total hip arthroplasty (THA): A retrospective single-center experience. Eur. J. Trauma Emerg. Surg..

[32] Stadler N., Lehner J., Trieb K. (2016). Prospective mid-term results of a consecutive series of a short stem. Acta Orthop. Belg..

[33] Aggarwal V.K., Elbuluk A., Dundon J., Herrero C., Hernandez C., Vigdorchik J.M., Schwarzkopf R., Iorio R., Long W.J. (2019). Surgical approach significantly affects the complication rates associated with total hip arthroplasty. Bone Jt. J..

[34] Graves S.C., Dropkin B.M., Keeney B.J., Lurie J.D., Tomek I.M. (2016). Does surgical approach affect patient-reported function after primary THA?. Clin. Orthop. Relat. Res..

[35] Klasan A., Neri T., Oberkircher L., Malcherczyk D., Heyse T.J., Bliemel C. (2019). Complications after direct anterior versus Watson-Jones approach in total hip arthroplasty: Results from a matched pair analysis on 1408 patients. BMC Musculoskelet Disord..

[36] Innmann M.M., Weiss S., Andreas F., Merle C., Streit M.R. (2016). Sports and physical activity after cementless total hip arthroplasty with a minimum follow-up of 10 years. Scand. J. Med. Sci. Sport..

[37] Lübbeke A., Zimmermann-Sloutskis D., Stern R., Roussos C., Bonvin A., Perneger T., Peter R., Hoffmeyer P. (2014). Physical activity before and after primary total hip arthroplasty: A registry-based study. Arthritis Care Res..

[38] Abe H., Sakai T., Nishii T., Takao M., Nakamura N., Sugano N. (2014). Jogging after total hip arthroplasty. Am. J. Sports Med..

[39] Swanson E.A., Schmalzried T.P., Dorey F.J. (2009). Activity Recommendations after Total Hip and Knee Arthroplasty. A Survey of the American Association for Hip and Knee Surgeons. J. Arthroplast..

[40] Ollivier M., Frey S., Parrate S., Flecher X., Argenson J. (2012). Does Impact Sport Activity Influence Total Hip Arthroplasty Durability?. Clin. Orthop. Relat. Res..

[41] Delasotta L.A., Rangavajjula A.V., Porat M.D., Frank M.L., Orozco F.R., Ong A.C. (2012). What Are Young Patients Doing After Hip Reconstruction?. J. Arthroplast..

[42] Bonnin M.P., Rollier J.C., Chatelet J.C., Ait-Si-Selmi T., Chouteau J., Jacquot L., Hannink G., Saffarini M., Fessy M.H. (2018). Can Patients Practice Strenuous Sports after Uncemented Ceramic-on-Ceramic Total Hip Arthroplasty?. Orthop. J. Sport Med..

